# Valve-Sparing Aortic Root Replacement Technique: Valsalva Graft versus Two Straight Tubular Grafts

**DOI:** 10.1155/2023/4076881

**Published:** 2023-02-03

**Authors:** Alexander Makkinejad, Bailey Brown, Rana-Armaghan Ahmad, Joanna Hua, Xiaoting Wu, Shinichi Fukuhara, Karen Kim, Himanshu Patel, G. Michael Deeb, Bo Yang

**Affiliations:** Department of Cardiac Surgery, Michigan Medicine, Ann Arbor, Michigan, USA

## Abstract

**Background:**

There are many variations in valve-sparing aortic root replacement techniques. Our aim is to determine the impact of the graft on mid-term outcomes: Valsalva graft vs. two straight tubular grafts.

**Methods:**

From 2004 to 2020, 332 patients underwent valve-sparing aortic root replacement with either a Valsalva graft (Valsalva group: *n* = 270) or two straight tubular grafts (two-graft group: *n* = 62). Data were obtained through chart review and the National Death Index. Primary outcomes were mid-term survival and freedom from reoperation.

**Results:**

The preoperative characteristics of the groups were similar, but the two-graft group had more type A dissections (32% vs. 19%) and emergent operations (26% vs. 15%) and was younger (45 vs. 50 years). Intraoperatively, the groups were similar, but the two-graft group had longer cross-clamp (245 vs. 215 minutes) and cardiopulmonary bypass times (284 vs. 255 minutes). Postoperative complications including reoperation for bleeding, stroke, pacemaker implantation, and renal failure were slightly more frequent in the Valsalva group, but the differences were not significant. Operative mortality was similar between the Valsalva and two-graft groups (0.7% vs. 0%). Five-year survival in the two-graft group was 100% compared to 96% in the Valsalva group (*p*=0.56). Five-year freedom from reoperation in the two-graft group was 100% compared to 93% in the Valsalva group (*p*=0.29).

**Conclusions:**

The Valsalva and two-graft techniques both have excellent short- and mid-term outcomes. The two-graft technique might have slightly better survival and freedom from reoperation, but a larger sample size and longer follow-up are needed to determine if these advantages are significant.

## 1. Introduction

For just over 30 years, valve-sparing aortic root replacement (VSARR) has been performed to treat aortic root aneurysms and aortic root dissection [1]. VSARR involves the replacement of all aortic sinuses and reattachment of the coronary arteries to the root prosthesis while preserving the native aortic valve. This procedure is particularly attractive to young patients because they can avoid long-term anticoagulant medications associated with mechanical valve replacement [2].

**Figure 1:**
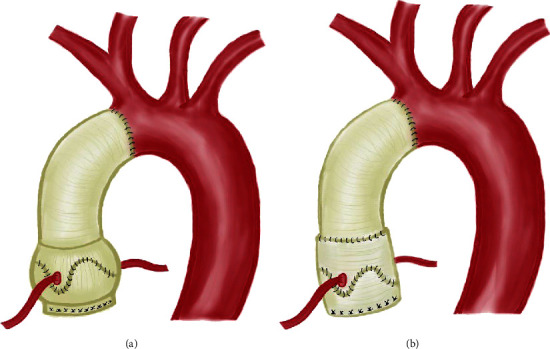
Illustration of valve-sparing aortic root and ascending aorta replacement with either a valsalva graft (David-V-Valsalva, (a)) or two straight tubular grafts (David-V-Smod, (b)).

The original VSARR can be generalized into two procedures—Yacoub remodeling and David reimplantation. The remodeling techniques orient the root prosthesis on top of the annulus, while the reimplantation techniques (David-I, IV, V, V-Smod) encapsulate the valve [3]. It is unknown which David reimplantation variation offers the best short- and long-term competence. More specifically, surgeons must choose between two types of Gelweave grafts (Terumo, Somerset, NJ), a Valsalva graft (David-V-Valsalva, Figure 1(a)), or two straight tubular grafts (David-V-Smod, Figure 1(b)). The Valsalva graft used in the David reimplantation procedure aims to reproduce the sinuses of Valsalva. Many surgeons have adopted the Valsalva graft and have reported satisfactory early and mid-term results in patients with and without connective tissue disorders [4–10]. The David-V-Smod procedure (two-graft technique), developed at Stanford in 2004, utilizes two straight tubular grafts: a large graft to create the neosinus and a small graft to create the sinotubular junction and replace the ascending aorta [11]. This technique is able to better accommodate each patient's unique aortic root anatomy and pathology, including the sizing of the neoannulus, pseudosinuses, sinotubular junction, and commissures [1]. Few studies exist assessing the clinical outcomes of this technique [12–14].

There is currently no literature directly comparing the short and mid-term clinical outcomes of patients undergoing VSARR using the David reimplantation Valsalva graft or two-graft techniques. We therefore present the perioperative and mid-term outcomes of patients undergoing David reimplantation with the Valsalva graft and two-graft techniques to treat aortic root aneurysm or acute type A aortic dissection. We hypothesize that the two-straight tubular grafts technique will provide similar short- and mid-term outcomes to the Valsalva graft technique.

## 2. Materials and Methods

The Institutional Review Board (IRB) or equivalent ethics committee of the University of Michigan, Michigan Medicine (Ann Arbor, MI) (HUM00155922, 12/2/2020), approved the study protocol and publication of data. Patient written consent for the publication of the study data was waived by the IRB due to minimal risk to patients.

### 2.1. Subjects

Between September 2004 and September 2020, 332 consecutive patients with aortic root aneurysm or acute type A aortic dissection underwent valve-sparing aortic root replacement with a Valsalva graft (*n* = 270) or two straight tubular grafts (*n* = 62). Data were obtained through the Society of Thoracic Surgery Data Warehouse to identify the relevant cohort and determine the perioperative, operative, and postoperative variables. These data were supplemented through medical chart review including echocardiographic details for specified variables. All derived data collected that support the findings of this study are available by request from the corresponding author. Survival and reoperation data were collected by medical record review and National Death Index data through June 30th, 2021 [15].

### 2.2. Operative Technique

The David procedure has been performed at our institution since late 2004 and consistently since 2005. The operative strategy at our institution for VSARR has been previously described [16]. Two modifications of the David procedure were used based on surgeon preference: Valsalva graft or two straight tubular grafts. One surgeon exclusively used the two-graft technique, and the other surgeons exclusively used the Valsalva graft technique. The David procedures were performed from 2004 to 2020, with 59% of these procedures performed from 2013 to 2020. The size of the Valsalva graft is determined by the left-noncommissural height. Six to twelve subannular stitches are placed. For the two-graft technique, the graft size of the neosinus is determined by the height of the cusps of the aortic valve, and the small graft for the neosinotubular junction reconstruction is determined by the neoannulus (basal ring of the aortic root). Eleven to thirteen subannular stitches are placed to prevent further dilation of the aortic root. The rims of dissected aortic sinus wall are anastomosed to the inside of the new sinus graft with full-thickness bites. Two coronary buttons are reimplanted. Hemiarch or arch replacement with reimplantation of one- to four-head vessels is performed based on the arch pathology. All procedures utilize cold crystalloid or blood cardioplegia and standard cardiopulmonary bypass. Antegrade cerebral perfusion or retrograde cerebral perfusion is used for cerebral protection during hypothermia circulatory arrest. One surgeon exclusively used antegrade cerebral perfusion whereas the other two surgeons used retrograde cerebral perfusion and profound hypothermia circulatory arrest for hemiarch and antegrade cerebral perfusion for more complex arch reconstruction.

### 2.3. Statistical Analysis

Continuous data were presented as median (interquartile range, 25%, 75%) and categorical data as *n* (%). Univariable comparisons between groups were performed using chi-square tests for categorical data. Wilcoxon rank-sum tests were performed for continuous data. Survival curves and long-term freedom from aortic insufficiency were estimated using the Kaplan–Meier method with the log-rank test. Cumulative incidence function curves were adjusted for death as a competing risk using the Fine and Gray subdistribution method to assess the incidence of reoperation due to severe aortic insufficiency over time. The Gray test was used to test the difference in the cumulative incidence function curves between groups. Cox proportional hazards model was used to evaluate risk factors for late mortality adjusting for VSARR technique, age, sex, bicuspid aortic valve, prior cerebrovascular accident, prior cardiac surgery, and primary indication. Follow-up rates were determined by using Clark's completeness index. *p* values less than 0.05 were considered statistically significant. Statistical calculations were performed with SAS (SAS Institute, Cary, NC).

## 3. Results

### 3.1. Demographics and Preoperative Data

Patient demographics were similar between the two-graft and Valsalva groups, except the two-graft group was younger (45 vs. 50 years). Both groups did not differ in diabetes mellitus (9.7% vs. 6.3%), coronary artery disease (18% vs. 19%), chronic lung disease (6.5% vs. 4.9%), hypertension (66% vs. 61%), congestive heart failure (8.1% vs. 3.3%), and previous cardiac surgery. The two-graft group had less aortic stenosis (0% vs. 6.3%) and more acute type A aortic dissections (32% vs. 19%) (Table 1).

### 3.2. Intraoperative Data

Intraoperatively, the two-graft group had fewer concomitant aortic valve procedures (4.8% vs. 15%), but more zone one (11% vs. 1.1%) and zone two (9.7% vs. 1.5%) arch replacement. The two-graft group had longer cardiopulmonary bypass (284 vs. 255 minutes) and cross-clamp (245 vs. 215 minutes) times. The average size of the implanted Valsalva graft was 30 mm. The mean size of the large and small grafts for the two-graft group was 34 and 24 mm, respectively, with a median large graft plication diameter of 29 mm (Table 2).

### 3.3. Perioperative Outcomes

There were no differences in perioperative outcomes between the two-graft and Valsalva groups, including operative mortality (0% vs. 0.7%). The incidence of complications such as reoperation due to bleeding or tamponade (0% vs. 1.9%), hemorrhagic stroke (0% vs. 2.2%), transient ischemic attack (0% vs. 0.8%), atrial fibrillation (27% vs. 31%), pneumonia (3.2% vs. 4.8%), and length of postoperative hospital stay (6 days vs. 6 days) were similar between the groups (Table 3).

### 3.4. Mid-Term Outcomes

**Figure 2:**
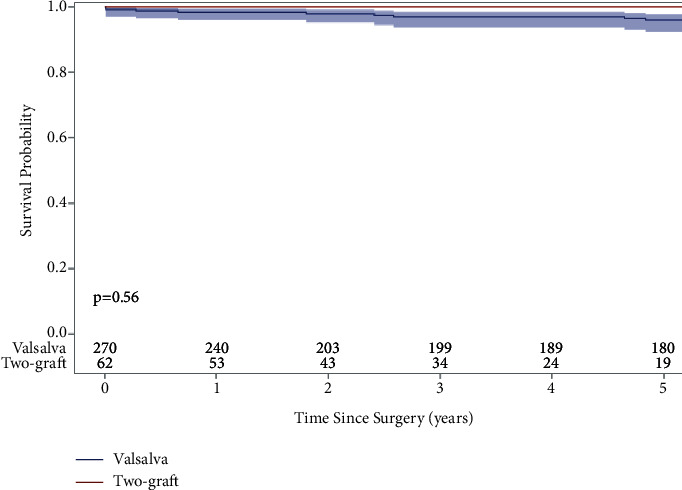
Kaplan–Meier long-term survival of valve-sparing aortic root replacement patients who underwent the David-V-Valsalva graft (5-year: 96%) or David-V-Smod (5-year: 100%) techniques.

The median follow-up time for mid-term survival was 6.4 years (3.2 years two-graft and 7.0 years Valsalva), and the follow-up rate for survival was 100% in both groups. The 5-year survival rate of the whole cohort was 97% (95% confidence interval: 94%, 98%). The 5-year survival rate in the two-graft group was 100%, and in the Valsalva group was 96% (Figure 2). Cox proportional hazards model adjusting for VSARR strategy, age, sex, bicuspid aortic valve, prior cerebrovascular accident, prior cardiac surgery, and a primary indication of operation showed that bicuspid aortic valve, prior cerebrovascular accident, and prior cardiac surgery were risk factors for late mortality (Table 4).

**Figure 3:**
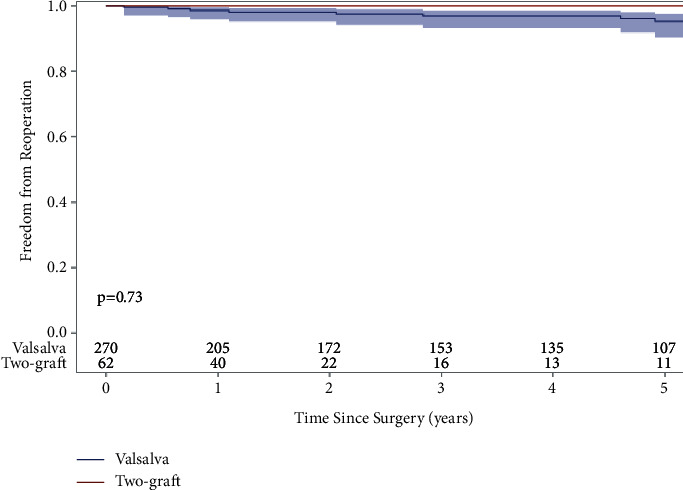
Freedom from reoperation for aortic insufficiency after valve-sparing aortic root replacement with either the David-V-Valsalva graft (5-year: 93%) or David-V-Smod (5-year: 100%) techniques showed no difference between the two implantation techniques (*p*=0.29).

The median follow-up time for mid-term reoperation data was 3.3 years (1.3 years two-graft and 4.0 years Valsalva), and the follow-up rate for reoperation was 65% in the two-graft group and 79% in the Valsalva group for the study period. The Valsalva group had a total of 13 reoperations due to severe aortic insufficiency, one reoperation due to acute type A aortic dissection, and one reoperation due to aortic root prosthetic graft infection while the two-graft group had no reoperations. The 5-year freedom from reoperation rate due to severe aortic insufficiency, adjusting for death as a competing factor, was 100% in the two-graft group and 93% in the Valsalva group (Figure 3).

**Figure 4:**
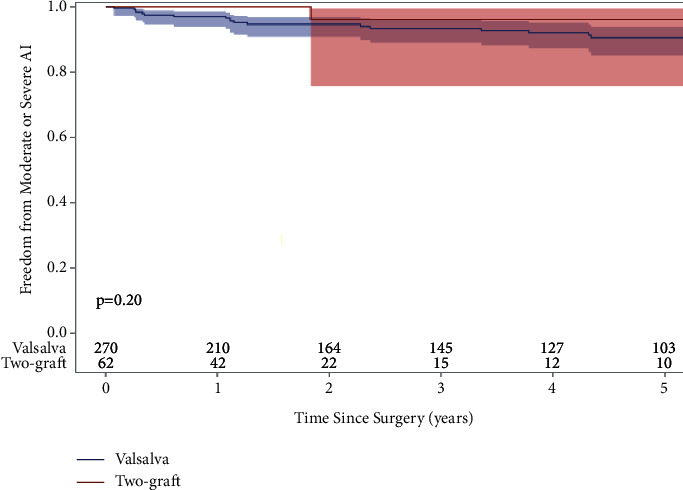
Kaplan–Meier freedom from moderate and severe aortic insufficiency of valve-sparing aortic root replacement patients who underwent David-V-Valsalva (5-year: 91%) or David-V-Smod (5-year: 96%) techniques (*p*=0.19).

The median follow-up time for echocardiographic data was 2.5 years (1.4 years two-graft and 3.5 years Valsalva), and the follow-up rate for echocardiographic data was 65% in the two-graft group and 79% in the Valsalva group for the study period. The 5-year freedom from aortic insufficiency grade moderate or higher, which was either detected clinically and confirmed by follow-up echocardiography, or found on routine yearly echocardiographic follow-up, was 96% in the two-graft group and was 91% in the Valsalva group (Figure 4).

## 4. Discussion

Our study is the first to directly compare the early and mid-term clinical outcomes of VSARR with David reimplantation using either the Valsalva graft or two-graft techniques. We report excellent outcomes for both techniques; there were no differences in postoperative complications in addition to no differences in operative, in-hospital, and 30-day mortalities between the two-graft and Valsalva groups (Table 1). There were also no differences in 5-year survival (two-graft: 100%, Valsalva: 96%), 5-year freedom from reoperation due to severe aortic insufficiency adjusting death as a competing factor (two-graft: 0%, Valsalva: 93%), and 5-year freedom from moderate or greater aortic insufficiency (two-graft: 96%, Valsalva: 91%) (Figures 1–3).

In 2000, De Paulis and colleagues introduced a new Dacron graft—the Valsalva graft—for the David reimplantation technique. The Valsalva graft skirt stretches in the horizontal plane to better recreate the sinotubular junction for a more natural aortic root anatomy. De Paulis et al. and colleagues reported exceptional early postoperative results and no operative deaths [17], and our study showed similar perioperative results (Table 3). In 2002, they utilized the Valsalva graft in the Bentall, reimplantation, and remodeling procedures and noted the Valsalva reimplantation technique decreased the risk of bleeding and stabilized the aortic wall and annulus [18]. Since De Paulis and colleagues' pioneering work, the majority of surgeons have adopted this VSARR technique to treat aortic root disease if the native aortic valve is salvageable. The Valsalva graft sinus distensibility has been shown to be appropriately preserved, and its valve-opening characteristics were consistent with normal valve function [19]. Excellent early outcomes in patients with and without Marfan syndrome have been reported [6–10]. The mid-term outcomes of VSARR with a Valsalva graft in our study were similar to those found in other large centers, such as the findings of a multicenter Italian study from 2006 which showed a 5-year freedom from moderate or higher aortic insufficiency of 89% [6], compared to 91% in our study.

**Figure 5:**
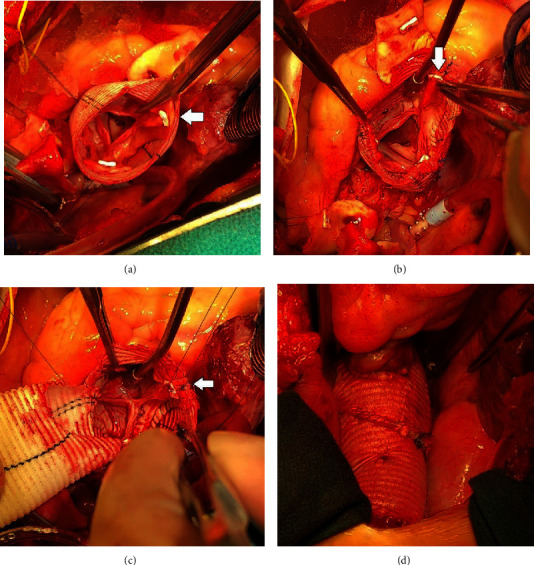
Example of a patient with a tall right-non commissural post (arrow) which is 2 cm taller than the left-right and the left-non commissural posts (a, b); anatomic variants such as this might be more easily accommodated when using the two-graft technique (c, d).

An additional David reimplantation technique, although far less commonly used due to its steep learning curve and unforgiving nature, is the David-V-Smod. The Stanford group reasoned that this technique allows the aortic valve to be more easily reimplanted within the larger graft, which recreates the neosinuses. Subsequently, the distal end of the smaller graft, which makes up the sinotubular junction and recreates the proximal ascending aorta, can be better matched to the diameter of the aorta than in the Valsalva reimplantation technique [10]. The advantage of the two-graft technique is that each implantation of the aortic root is tailored to each individual patient's specific anatomy instead of a one-size fits all approach. For example, the heights of the various commissural posts can be different, and sometimes, one commissural post can be as much as 1–2 cm higher than the other two (Figures 5(a) and 5(b)). It is easier to utilize the two-graft technique to accommodate this uneven commissural height (Figures 5(c) and 5(d)). The Valsalva graft has fixed dimensions. For example, the 30 mm Valsalva graft has a 30 mm annulus, 30 mm sinotubular junction, and 30 mm height of the skirt portion of the graft. The surgeons must fit the Valsalva graft with the patient's aortic valve. On the other hand, the two-graft technique uses the graft to fit the patient's aortic valve. Although there was a trend of better short- and mid-term outcomes with the two-graft technique, these differences were not significant, most likely due to the small sample size of the two-graft group. A small number of aortic centers have adopted this Stanford modification, and very few studies have demonstrated its clinical outcomes. Similar to our findings, Kvitting and colleagues at Stanford reported excellent perioperative outcomes of 233 patients undergoing the David-I, David-V, and David-V-Smod procedures with 188 of these patients undergoing the David-V-Smod [13]. Their study reported a 5- and 10-year survival rate of 98.7% and 93.5%, 5- and 10-year freedom from reoperation of 98.0% and 92.2%, and 5- and 10-year freedom from greater than 2+ aortic regurgitation was 97.4% and 95.3%, respectively. We report exceptional but not different mid-term survival between the Valsalva implantation and David-V-Smod techniques. The Valsalva graft 5-survival was 96%, and similar findings have been previously reported, including the results of De Paulis et al. in their multicenter study of four Italian centers [4–6] (Figure 2). Hospital Universitaria 12 Octubre group reported a 5-year survival of 97% for 120 patients who received the David-V-Smod, and our 5-year survival with this technique was 100% [12].

A common determinant for reoperation of VSARR procedures is the postoperative progression of aortic insufficiency after the initial VSARR surgery. Thus, we found that the David-V may offer better hemodynamics as we report a 5-year freedom from moderate or severe aortic insufficiency of 96% and 91% in the two-graft and Valsalva groups, respectively, although this difference was not significant (Figure 4). The Valsalva graft has been effective in patients with and without Marfan syndrome; the reported 10-year freedom from moderate or severe aortic insufficiency in patients with Marfan syndrome is 93.5% and without Marfan syndrome is 87.1% [5]. These data are lacking for the David-V-Smod procedure.

Reoperation between both techniques was also not different in our study, confirming previous reports. Similar to our results for the Valsalva group, studies have reported the 5-year freedom from reoperation due to aortic insufficiency ranges from 89–91%, and De Paulis et al. reported an overall 10-year freedom from reoperation of 91% [4–7] (Figure 3). It seems that the David-V-Smod may offer slightly better reoperation rates, however, as the Hospital Universitaria 12 Octubre group reported a 5-year freedom from reoperation of 96% [12], and Kvitting et al. reported a 10-year freedom from reoperation for all causes of 92.2% [13]. The 5-year freedom from reoperation rates due to severe aortic insufficiency for the David-V-Smod group was 100% in our study (Figure 3). Overall, our findings are consistent with the literature and demonstrate the efficacy of both procedures and their successful implementation at different institutions.

On average, patients undergoing the David-V-Smod were on cardiopulmonary bypass and cross-clamped 29 and 30 minutes longer, respectively, than those who received the Valsalva reimplantation. This was due to more complex operations in the two-graft group, including more acute type A dissection repair and more aggressive arch replacement. Although short- and mid-term results are excellent for both the Valsalva reimplantation and David-V-Smod, there is far less experimental and clinical evidence for the two-graft technique; however, our study demonstrated that the two-graft technique may have exhibited mildly better survival, reoperation, and lower incidence of moderate or severe aortic insufficiency. Thus, a longer follow-up is required to determine whether this difference is significant in the long term.

### 4.1. Limitations

This study is limited as a retrospective cohort study—the choice of Valsalva graft versus two straight tubular grafts was by surgeon preference and not randomized, creating some differences between the groups. Because of the relatively low sample size of the two-graft group, it was difficult to control for some important preoperative factors including age and incidence of acute type A aortic dissection as the primary indication. That being said, the fact that the two-graft group had significantly more aortic dissection patients than the Valsalva group, while still having similar midterm outcomes, shows that at the very least, the two-graft technique can be safely used in a more high-risk set of patients with comparable results to the Valsalva graft. The National Death Index database was combined with a manual chart review to assess for long-term mortality, but this method may not catch 100% of deaths. Our institution aims to conduct follow-up yearly echocardiograms in aortic valve replacement patients, but we are not able to get full echocardiographic follow-up data for all patients. Lastly, although our institution is one of the few tertiary health centers in its area and we therefore expect most of our patients requiring reoperation would return to our institution, some patients may have had reoperations at outside hospitals, which could have caused us to underestimate reoperation rates.

## 5. Conclusion

The mid-term clinical outcomes of both techniques of valve-sparing aortic root replacement with David reimplantation are excellent, including survival and freedom from reoperation. Long-term results are needed to determine how the newer two-graft technique compares to the Valsalva graft technique.

## Figures and Tables

**Table 1:** Preoperative and demographic data.

Variable	Valsalva (*n* = 270)	Two-graft (*n* = 62)	*p* value
Patient age (years)	50 (40, 58)	45 (32, 53)	0.02
Sex ((%) female)	41 (15)	9 (15)	0.89
Body surface area (m^2^)	2.15 (2.00, 2.34)	2.24 (2.04, 2.45)	0.22
Diabetes mellitus	17 (6.3)	6 (9.7)	0.40
CAD	50 (19)	11 (18)	0.89
MI (<30 days)	17 (6.3)	7 (11)	0.18
Chronic lung disease	13 (4.9)	4 (6.5)	0.54
Hypertension	166 (61)	41 (66)	0.50
Renal failure on dialysis	2 (0.7)	0 (0)	>0.99
Creatinine (mg/dL)	1.0 (0.8, 1.1)	1.0 (0.9, 1.2)	0.51
Arrhythmia	63 (23)	9 (15)	0.13
CHF	9 (3.3)	5 (8.1)	0.15
BAV	19 (7.0)	4 (6.5)	>0.99
Marfan syndrome	54 (20)	18 (30)	0.11
Loeys-Dietz syndrome	1 (0.4)	1 (1.6)	0.34
Aortic insufficiency			0.08
None	61 (23)	17 (27)	0.42
Trace/trivial	42 (16)	10 (16)	0.91
Mild	73 (27)	14 (23)	0.47
Moderate	41 (15)	13 (21)	0.27
Severe	53 (20)	8 (13)	0.22
Previous cardiac surgery	12 (4.4)	0 (0)	0.13
Aortic valve procedure^*∗*^	1 (0.4)	0 (0)	>0.99
Mitral valve procedure	6 (2.3)	0 (0)	0.60
Tricuspid valve procedure	0 (0)	0 (0)	>0.99
Ascending aorta/arch procedure	3 (1.1)	0 (0)	>0.99
Coronary artery bypass grafting	2 (0.4)	0 (0)	>0.99
NYHA class			0.16
I	27 (10)	12 (19)	0.04
II	23 (8.5)	2 (3.2)	0.19
III	7 (2.6)	1 (1.6)	>0.99
IV	1 (0.4)	1 (1.6)	0.34
Ejection fraction			
>60%	77 (29)	20 (32)	0.56
40–60%	167 (62)	35 (56)	0.43
20–40%	14 (5.2)	2 (3.2)	0.75
Year of surgery			<0.001
2004–2010	91 (34)	1 (2)	
2011–2015	101 (37)	20 (32)	
2016–2020	78 (29)	41 (66)	

Data presented as median (interquartile range) for continuous variables and number (percentage) for categorical variables. Abbreviations: CAD, coronary artery disease; MI, myocardial infarction; CHF, congestive heart failure; BAV, bicuspid aortic valve; CABG, coronary artery bypass grafting. ^*∗*^1 patient in the Valsalva group underwent previous aortic valve repair (resuspension).

**Table 2:** Operative data.

Variable	Valsalva (*n* = 270)	Two-graft (*n* = 62)	*p* value
Primary indication			0.02
Root aneurysm	220 (81)	42 (68)	
Acute type A aortic dissection	50 (19)	20 (32)	
Timing of operation			0.12
Elective	210 (78)	41 (66)	0.05
Urgent	19 (7.0)	5 (8.1)	0.79
Emergent/salvage	41 (15)	16 (26)	0.05
Concomitant procedure			
CABG	12 (4.5)	2 (3.2)	0.66
Mitral valve surgery	15 (5.6)	4 (6.5)	0.76
Tricuspid valve surgery	4 (1.5)	2 (3.2)	0.31
Aortic valve repair	41 (15)	3 (4.8)	0.03
Ascending aorta procedure	233 (86)	49 (79)	0.15
Hemiarch replacement	60 (22)	8 (13)	0.10
Total arch replacement	4 (1.5)	2 (3.2)	0.31
Zone 1 replacement	3 (1.1)	7 (11)	<0.001
Zone 2 replacement	4 (1.5)	6 (9.7)	0.004
Zone 3 replacement	2 (0.7)	2 (3.2)	0.16
CPB time	255 (233, 284)	284 (265, 346)	<0.001
Cross-clamp time	215 (195, 238)	245 (228, 278)	<0.001
PRBC (units)	0.0 (0.0, 2.0)	0.0 (0.0, 1.0)	0.16
Size of implant			
Valsalva graft (mm)	30 (30, 32)		
Large graft (mm)		34 (34, 34)	
Plication of large graft (mm)		29 (29, 30)	
Small graft (mm)		24 (24, 26)	

Data presented as median (interquartile range) for continuous variables and number (percentage) for categorical variables. Abbreviations: CABG, coronary artery bypass grafting; CPB, cardiopulmonary bypass; PRBC, packed red blood cells.

**Table 3:** Perioperative data.

Variable	Valsalva (*n* = 270)	Two-graft (*n* = 62)	*p* value
Ventilator time (hours)	7 (4, 14)	5 (3, 11)	0.13
Re-operation for bleeding/tamponade	5 (1.9)	0 (0)	0.59
Thoracotomy	1 (0.4)	0 (0)	>0.99
Delayed sternal closure	1 (0.4)	0 (0)	>0.99
Sternal dehiscence	5 (1.9)	0 (0)	0.59
Postoperative blood units	0.0 (0.0, 1.0)	0.0 (0.0, 0.0)	0.03
Hemorrhagic stroke	6 (2.2)	0 (0)	0.60
TIA	2 (0.8)	0 (0)	>0.99
Coma	1 (0.4)	0 (0)	>0.99
Atrial fibrillation	85 (31)	17 (27)	0.53
Complete heart block or pacemaker	5 (1.9)	0 (0)	0.59
New onset renal failure	5 (2.0)	0 (0)	0.59
Prolonged ventilation	29 (11)	5 (8.1)	0.53
Pneumonia	13 (4.8)	2 (3.2)	0.75
Cardiac arrest	3 (1.1)	0 (0)	>0.99
Gastrointestinal event	13 (4.8)	1 (1.6)	0.48
Multisystem organ failure	1 (0.4)	0 (0)	>0.99
MI	1 (0.4)	0 (0)	>0.99
Hospital stay (days)	6 (5, 8)	6 (5, 7)	0.90
In-hospital mortality	2 (0.7)	0 (0)	>0.99
30-day mortality	2 (0.7)	0 (0)	>0.99
Operative mortality^*∗*^	2 (0.7)	0 (0)	>0.99

Data presented as median (interquartile range) for continuous variables and number (percentage) for categorical variables. ^*∗*^Operative mortality is based on the society of thoracic surgeons definition and includes all deaths, regardless of cause, occurring during the hospitalization in which the operation was performed, even if after 30 days (including patients transferred to other acute care facilities); and all deaths, regardless of cause, occurring after discharge from the hospital, but before the thirtieth postoperative day. Abbreviations: TIA, transient ischemic attack; MI, myocardial infarction.

**Table 4:** Cox proportional hazards regression for the risk factors of long-term mortality.

Variable	Hazard ratio (95% CI)	*p* value
VSARR technique (two-graft vs. valsalva)	0.65 (0.08, 5.29)	0.69
Age	0.98 (0.94, 1.02)	0.37
Gender (male vs. female)	2.59 (0.33, 20.38)	0.37
Bicuspid aortic valve	1.88 (1.17, 3.02)	0.01
Prior cerebrovascular accident	9.04 (1.67, 48.83)	0.01
Prior cardiac surgery	3.43 (1.13, 10.37)	0.03
Primary indication (dissection vs. aneurysm)	1.63 (0.48, 5.57)	0.44

Abbreviations: VSARR, valve-sparing aortic root replacement.

## Data Availability

Raw data were generated at Michigan Medicine, University of Michigan, Ann Arbor, MI. Derived data supporting the findings of this study are available from the corresponding author on request.
